# Supplementary material for: MetaRibo-Seq measures translation in microbiomes
Source: Nat Commun. 2020 Jun 29;11:3268. doi: 10.1038/s41467-020-17081-z (PMC7324362; doi:10.1038/s41467-020-17081-z)
Supplement: Supplementary file 10 — Supplementary Data 7 [file 41467_2020_17081_MOESM10_ESM.zip › File2/Confidence_High_Taxonomy/286448_out.krona.html]

Javascript must be enabled to view this page.

members
magnitude
magnitudeUnassigned
count
unassigned
taxon
rank

286448\_out

1

1
2759
superkingdom

1
kingdom
4751

1
subkingdom
451864

1
phylum
4890

1
147538
subphylum

1
147548
class

1
order
5120

1
34371
family

1
5121
genus

1

SRS1041145\_contig\_number\_13031
225359
species
